# CRISPR-engineered mosaicism rapidly reveals that loss of *Kcnj13* function in mice mimics human disease phenotypes

**DOI:** 10.1038/srep08366

**Published:** 2015-02-10

**Authors:** Hua Zhong, Yiyun Chen, Yumei Li, Rui Chen, Graeme Mardon

**Affiliations:** 1 Department of Pathology and Immunology, Baylor College of Medicine, Houston, TX 77030; 2 HGSC, Baylor College of Medicine, Houston, TX 77030; 3 Department of Molecular and Human Genetics, Baylor College of Medicine, Houston, TX 77030; 4 Program in Departmental Biology, Baylor College of Medicine, Houston, TX 77030; 5 Department of Neuroscience, Baylor College of Medicine, Houston, TX 77030; 6 Department of Ophthalmology, Baylor College of Medicine, Houston, TX 77030

## Abstract

The era of genomics has demanded the development of more efficient and timesaving approaches to validate gene function in disease. Here, we utilized the CRISPR-Cas9 system to generate *Kcnj13* mutant mice by zygote injection to verify the pathogenic role of human *KCNJ13*, mutations of which are thought to cause Leber congenital amaurosis (LCA), an early-onset form of blindness. We found that complete loss of *Kcnj13* is likely postnatal lethal. Among surviving F0-generation mice examined, 80% show mosaic KCNJ13 expression in the retinal pigment epithelium (RPE). Mosaic expression correlates with decreased response to light and photoreceptor degeneration, indicating that *Kcnj13* mutant mice mimic human *KCNJ13*-related LCA disease. Importantly, mosaic animals enable us to directly compare *Kcnj13* mutant and wild-type RPE cells in the same eye. We found that RPE cells lacking KCNJ13 protein still survive but overlying photoreceptors exhibit cell degeneration. At the same time, wild-type RPE cells can rescue neighboring photoreceptor cells that overlie mutant RPE cells. These results suggest that KCNJ13 expression is required for RPE cells to maintain photoreceptor survival. Moreover, we show that CRISPR-Cas9 engineered mosaicism can be used to rapidly test candidate gene function *in vivo*.

With the development of high-throughput sequencing technologies, more and more candidate disease genes are rapidly being identified in human patients. Therefore, it is crucial to develop efficient and timesaving approaches to test pathogenicity and further study the molecular mechanism by which each candidate gene functions. Recently, clustered regularly interspaced short palindromic repeats (CRISPR)-associated (Cas) protein 9 system (known as CRISPR-Cas9) has been demonstrated as an efficient and simple genome-editing tool^1^,^2^,^3^,^4^,^5^,^6^. The CRISPR-Cas9 system consists of Cas9 endonuclease and a single-guide RNA (sgRNA). The sgRNA contains a targeting guide sequence that can direct Cas9 endonuclease to cleave target DNA and introduce site-specific double-stranded breaks (DSBs). DSBs in mammalian DNA can be repaired by non-homologous end joining, leaving random insertions and deletions, or by homologous recombination, resulting in precise sequence editing. By zygote injection of sgRNA and *Cas9* mRNA with or without donor DNA, researchers have consistently achieved expected DNA editing efficiently and rapidly in the mouse^5^,^6^,^7^. The CRISPR-Cas9 system has been successfully applied to test relationships between mutations and diseases by introducing new mutations to generate disease phenotypes^8^,^9^ or by correcting existing mutations to rescue disease phenotypes^10^,^11^,^12^,^13^.

*KCNJ13*, encoding inwardly rectifying potassium channel subunit Kir7.1, is expressed at the apical side of the retinal pigment epithelium (RPE)^14^,^15^,^16^. Mutations in *KCNJ13* are associated with Leber congenital amaurosis (LCA)^17^, which is clinically characterized by severe and early visual loss, sensory nystagmus, amaurotic pupils, fundus changes and minimal or absent electrical signals on electroretinogram (ERG)^18^. KCNJ13 is likely to regulate K^+^ transport and the electrical response of the RPE to light-evoked changes in sub-retinal K^+^ concentration^16^. However, the retinal pathogenesis caused by loss of *KCNJ13* function has not been explored. To understand the etiology of *KCNJ13*-mediated LCA, a *Kcnj13* mouse model would be of great value; however, such a model has not been reported. Mouse and human KCNJ13 proteins share 93% identity and have homologous expression patterns in RPE cells. Therefore, as has been the case for virtually all other LCA disease genes^18^, *Kcnj13* mutant mice are likely to mimic the pathogenesis of human *KCNJ13* LCA and would be a very useful model to investigate the molecular basis of and develop targeted therapies for LCA disease.

Here, the CRISPR-Cas9 system was used to generate mouse *Kcnj13* null alleles by zygote injection of sgRNA and *Cas9* mRNA designed to target the *Kcnj13* start codon since a homozygous nonsense mutation has been identified in *KCNJ13* in one LCA patient^17^. As expected, many F0-generation (F0) mice have *Kcnj13* null allele(s). However, after germline transmission of a *Kcnj13* null allele, all F1 and F2 mice possessing two *Kcnj13* null alleles die at postnatal day 1 (P1). To date, homozygous lethal alleles have required researchers to adopt the *Cre-loxP* system to produce conditional loss-of-function mosaics in which deletion of the gene of interest is limited to only the cell types or tissues of interest. This approach enables animals to survive beyond the lethal phase but at the same time permits study of the null phenotype in specific groups of cells. However, recent reports indicate that compound mosaicism, where substantial numbers of cells in F0 animals carry mutations in both copies of a gene, can be detected in CRISPR-Cas9 generated mice^6^,^7^,^19^. The frequency of such mosaics and their utility as a disease model has not been reported. In this study, we examined 10 surviving F0 mutant mice to investigate the incidence of CRISPR-Cas9 produced mosaicism. Interestingly, 80% (8/10) of F0 mice displayed KCNJ13 mosaic expression in retinal sections. The expression pattern and matched morphological analysis rapidly revealed that loss of *Kcnj13* function causes photoreceptor degeneration. In addition, we observe that RPE cells lacking KCNJ13 protein still survive and that wild-type RPE cells can rescue neighboring photoreceptor cells that overlie mutant RPE cells, indicating that KCNJ13 expression is required for RPE cells to maintain photoreceptor survival. Our results demonstrate that CRISPR-Cas9 generated mosaicism can be efficiently utilized to overcome early lethality and subsequently perform phenotypic analysis and dissect gene function in disease by comparing mutant and wild-type cells in the same F0 animal.

## Results

### Generation of *Kcnj13* mutant mice by zygote injection

**Figure 1:**
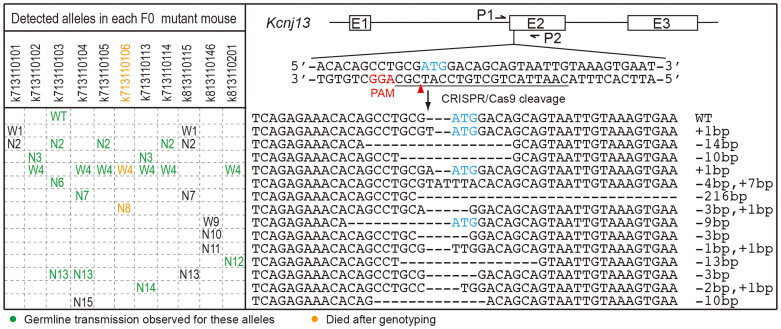
CRISPR-Cas9-induced mutations in *Kcnj13* F0 mice. E1, E2 and E3 in the schematic of the *Kcnj13* gene structure indicate three known exons. P1 and P2 are genotyping primers for the CRISPR-Cas9 targeting site. The blue ATG is the putative start codon of *Kcnj13*. The red arrowhead indicates the potential Cas9 cleavage site. PAM: the protospacer-adjacent motif required for the binding and cleavage of DNA by CRISPR-Cas9. The underlined sequence is the sgRNA target we selected to delete the start codon. Small deletions (1–14 bp), small insertions (1 and 7 bp), and one large deletion (216 bp) were detected. All ATG-deleted alleles are defined as candidate *Kcnj13* null alleles (N, named N2-15); alleles that leave the ATG intact are defined as wild-type alleles (W, named W1, W4 and W9). The wild-type reference allele is abbreviated as “WT”. Seven F0 mutant mice were selected to intercross or cross with wild-type C57BL/6 mice to test if their mutant alleles could be transmitted to F1 animals; transmitted alleles are labeled in green.

To make a *Kcnj13* null allele, the start codon was selected as the target site of CRISPR-Cas9 cleavage (Fig. 1; Supplementary Fig. S1a). Two concentrations of an sgRNA and *Cas9* mRNA cocktail (50 ng/µl + 100 ng/µl; 12.5 ng/µl + 25 ng/µl) were used to perform zygote injection (Supplementary Fig. S1b) according to reported injection dosages^5^,^7^. The high concentration generates mutant mice with higher efficiency while the low concentration can minimize potential toxicity and off-target effects caused by the high dosage^20^. From a high-concentration injection, 20 newborns died at P1 and only eight survived. All eight surviving F0 founder mice and genotyped deceased mice had mutations at the CRISPR-Cas9 targeted site as assayed by a PCR-RFLP test (Supplementary Fig. S1a,c). In contrast, 38/39 of newborns survived low-concentration injections and only three exhibited expected mutations. Furthermore, PCR sub-cloning and DNA sequencing was performed using tail-derived genomic DNA from all 11 surviving F0 founder mice carrying targeted mutations detected by PCR-RFLP. Eight clones were sequenced for each F0 survivor. All animals showed at least two mutated alleles each (Fig. 1). All ATG-deleted alleles are considered as candidate *Kcnj13* null alleles (N, named N2-N15 for convenience); three alleles that leave the ATG intact but insert 1 bp or delete 12 bp immediately 5′ of the ATG are referred to as wild-type alleles (W, named W1, W4 and W9). Except for k713110103, each surviving F0 mouse carried at least one “W” allele among sequenced clones.

### Potential lethality of *Kcnj13* null allele

Seven F0 mutant mice were selected to intercross or cross with wild-type C57BL/6 mice. With the exception of the N15 allele, all mutant alleles detected in tail DNAs from these seven F0 mice were transmitted to the next (F1) generation. In addition, germline transmission of a wild-type allele was also detected in progeny of F0 mouse k713110103 (Fig. 1, transmitted alleles are marked in green). All F1 pups with the genotype N/W4 or W4/W4 survive, while F1 pups with the genotype N/N die at P1, indicating that mice homozygous or transheterozygous for candidate *Kcnj13* null alleles are postnatal lethal. To further confirm the lethality, F1 pups with the genotype N/WT from crosses of F0 mutant to C57BL/6 were intercrossed and resulting F2 pups with the N/N genotype also died at P1. Potential off-target effects as a cause for this lethality were not systematically investigated in this study although transheterozygotes of independently derived null alleles also die at P1.

### KCNJ13 mosaic expression and functional defects in F0 mutant mice

Homozygous mutations in the human gene *KCNJ13* are associated with LCA disease and impairment of rod and cone ERGs^17^. However, since we are unable to study postnatal eye development or function in *Kcnj13* null mice due to P1 lethality, we considered producing a conditional loss-of-function (*Cre-loxP*) allele. Unfortunately, it takes more than one year to generate the correct genotype (*gene^floxP^/gene^floxP or null^; Cre/+*) for such analyses, including introduction of *loxP* sites in the genome using gene targeting in ES cells. However, chimeric and mosaic analyses are powerful tools for dissecting gene function in development and disease, especially for lethal genes^21^. Since tail DNA from our F0 mutant mice exhibited mosaicism (more than two alleles in one individual, Fig. 1) and cells with genotypes N/N, N/W, and/or W/W likely co-exist in one animal. We therefore expected to observe mosaic KCNJ13 expression in RPE cells and associated morphological defects in the retina. Three different time points were selected to test dark-adapted ERG a- and b-wave amplitudes followed by histological and immunohistochemical analyses of retinal morphology and KCNJ13 expression in the RPE cell layer.

**Figure 2:**
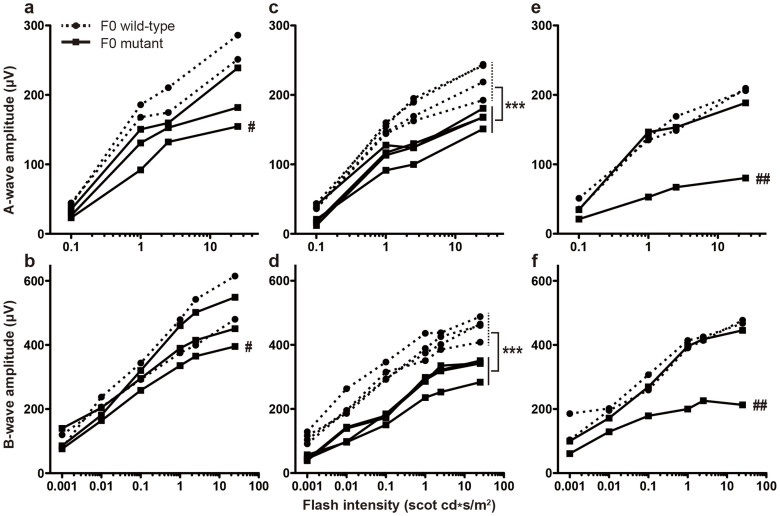
Decreased responses to light in CRISPR-Cas9 F0 mutant mice as measured by dark-adapted ERG tests. ERGs were tested at 13 weeks (a,b), 22 weeks (c,d), and 33 weeks (e,f) of age. ‘#’ indicates F0 mutant mouse k813110115; ‘##’ indicates F0 mutant mouse k713110104. ‘***’ indicates a two-way ANOVA *p*-value < 0.001. Compared to F0 wild-type mice, decreased ERG a- and b-waves suggest functional deficiencies in F0 mutant retinas.

**Figure 3:**
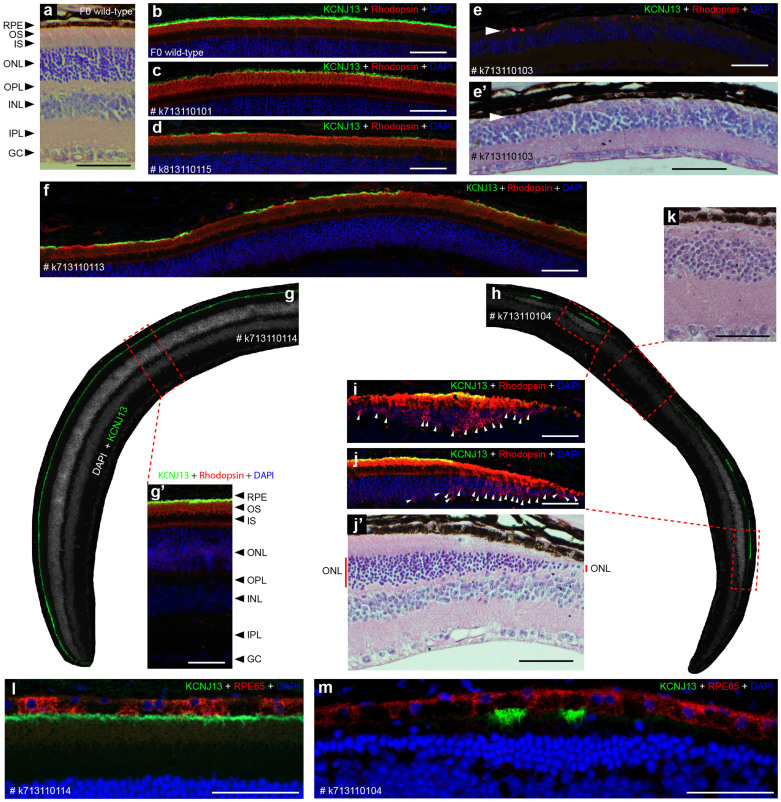
CRISPR-Cas9-induced F0 mosaic loss of KCNJ13 in RPE cells causes photoreceptor disease phenotypes. Protein expression of KCNJ13 is shown in green, rhodopsin and RPE65 are shown in red. DAPI (blue) marks nuclei. The loss of photoreceptor cells was determined by comparing the thickness of the ONL between mutant and wild-type mice. (a) A cross-section of a F0 wild-type eyecup stained with H&E is shown. (b) KCNJ13 expression in wild-type RPE cells and rhodopsin localization in the outer segment (OS) of photoreceptor cells are shown. Images a and b are from adjacent serial sections of the same F0 wild-type eyecup prepared from an animal at 13 weeks of age. (c,d,e,f,g,h) KCNJ13 mosaic expression and rhodopsin localization in retinas prepared from animals at 13 weeks (c,d), 17 weeks (e), 22 weeks (f), and 33 weeks of age (g,h). Each image represents one mosaic mutant mouse (each unique ID is included with each image). (i,j,k) Loss of photoreceptor cells (i,k) and accompanied rhodopsin mislocalization, indicated by white arrowheads (i,j). Images e and e’, as well as j and j’, are taken from similar regions of adjacent serial sections. k and j’ are from the same H&E stained section and show that retinal morphology and loss of photoreceptor cells correlates with regions lacking KCNJ13 protein. (l,m) Co-staining of KCNJ13 and RPE65 to demonstrate that mutant RPE cells are surviving. RPE, retinal pigment epithelium. OS, outer segment. IS, inner segment. ONL, outer nuclear layer. OPL, outer plexiform layer. INL, inner nuclear layer. IPL, inner plexiform layer. GC, ganglion cell. Scale bar is 50 µm.

At 13 weeks of age, three F0 mutant mice and two F0 wild-type mice were tested. One of the three F0 mutant mice showed decreased a- and b-waves compared to F0 wild-type mice (k813110115, marked by a hash key, Fig. 2a,b). After ERG testing, these five mice were sacrificed and eyecups prepared for immunofluorescence (IF) staining and hematoxylin and eosin (H&E) staining. No gross morphological changes were detected in mutant retinas; however, two F0 mutant mice (k713110101 and k813110115) exhibited mosaic KCNJ13 expression (Fig. 3c,d; Supplementary Fig. S2b) compared to F0 wild-type mice (Fig. 3b; Supplementary Fig. S2a). In particular, mouse k813110115 showed patchy KCNJ13 expression with corresponding rhodopsin mislocalization in the regions lacking KCNJ13 (Supplementary Fig. S2b), which may explain the clearly decreased ERG a- and b-waves in this animal^22^. To further investigate these phenotypes, ERGs were tested and morphological examinations performed on 22 and 33 week old mice. In our 22-week-old F0 mouse cohort (four F0 wild-type and four F0 mutant), all mutant mice showed significantly decreased ERG a- and b-waves (*p*-value < 0.001, Fig. 2c,d). Consistently, all mutant mice displayed mosaic KCNJ13 expression (mouse k713110113 is shown as a representative example in Fig. 3f and Supplementary Fig. S2c), indicating that patches of RPE cells completely lose *Kcnj13* function; however, complete loss of KCNJ13 expression in large patches of the RPE was not observed, and no significant morphological changes were observed in the 22-week-old cohort. In 33-week-old F0 mice (two F0 wild-type and two F0 mutant), one mutant mouse showed greatly decreased ERG a- and b-waves (k713110104, marked by double hash keys, >50%, Fig. 2e,f) while another mutant mouse (k713110114) had ERG results similar to F0 wild-type mice. In all cases, ERG results were consistent with our observed KCNJ13 mosaic expression. As shown in Fig. 3g and 3g’, KCNJ13 expression in mouse k713110114 is very similar to wild-type (Fig. 3b) while mouse k713110104 showed only a few small patches of KCNJ13-expressing RPE cells (Fig. 3h), which correlated well with severe photoreceptor degeneration (Fig. 3k). We noticed that a relatively large patch of KCNJ13 expressing cells can rescue survival of surrounding photoreceptors although some photoreceptor cells show rhodopsin mislocalization near the boundary between KCNJ13 expressing and non-expressing cells (Fig. 3i). In addition, severe rhodopsin mislocalization (Fig. 3j, marked by white arrowheads) and thinning of the outer nuclear layer (ONL, Fig. 3j’, marked by a solid red line) are observed where KCNJ13 expression is absent while normal rhodopsin localization and thicker ONL are observed where KCNJ13 is present. These observations indicate that *Kcnj13* mutant RPE cells are associated with rhodopsin mislocalization and photoreceptor degeneration and that KCNJ13 expression is required for RPE cells to maintain photoreceptor cell survival.

To test if loss of KCNJ13 expression causes loss of RPE cells, we first examined *Kcnj13* mutant RPE cells by co-staining for KCNJ13 and RPE65, an RPE cell marker which is abundantly expressed in RPE cells^23^,^24^. As shown in Fig. 3l and 3m, we detect RPE65 expression in both KCNJ13 expressing and non-expressing RPE cells, including those accompanied by severe loss of photoreceptors (Fig. 3m). Second, we tested for potential RPE cell death by TUNEL staining of retinal sections from mosaic mouse k713110104 and did not observe any TUNEL-positive RPE cells, while TUNEL-positive photoreceptor cells overlying mutant RPE cells were occasionally observed (Supplementary Fig. S3). These two lines of evidence suggest that KCNJ13 expression in RPE cells is required for photoreceptor survival.

### Severe retinal defects in F0 mosaic mice with extensive loss of *Kcnj13* function

One F0 mutant mouse (k713110103) was sacrificed at 17 weeks of age for histological and immunofluorescence evaluation. As shown in Fig. 3e and 3e’, photoreceptor cells in this mouse are almost entirely absent with only a few surviving (marked by a white arrowhead). Consistent with the near complete loss of photoreceptors, KCNJ13 expression is not detected in serial sections covering half of each eyecup. We hypothesized that the F0 mutant mice (k713110103 and k713110104) showing significant retinal defects likely have a high frequency of *Kcnj13* mutant cells. We therefore performed additional PCR sub-cloning and sequencing (Table 1) to determine the genotype of retinal sections lacking KCNJ13 expression. F0 mutant mouse k713110114, which exhibited KCNJ13 expression and ERGs similar to wild-type mice, was used as a control. Consistent with the observed lack of KCNJ13 protein, all sequenced clones (n = 155) of PCR products from k713110103 tail and retinal section DNA are indeed *Kcnj13* null alleles. Thus, although germline transmission of a wild-type allele was observed for this mouse, which may explain its ability to survive past P1, the vast majority of tail and retinal tissue tested appears to be homozygous/transheterozygous mutant. For k713110104 retinal section DNA, only 4.3% of sequenced clones are wild-type allele W4, consistent with the observed sporadic expression of KCNJ13 (Fig. 3h). For k713110114 tail and retinal section DNA, the frequencies of wild-type (W4) and null (N2) alleles are almost equal and no other mutant allele was found. These sequencing results suggest that strong retinal defects observed in F0 mosaic mice result from a high frequency of *Kcnj13* homozygous or transheterozygous null mutant RPE cells.

To test the possibility that off-target effects possibly accompanying *Kcnj13* mutations are the cause of the retinal degeneration observed, we performed a series of Surveyor assays on 12 predicted off-target sites (Supplementary Fig. S4a). Based on an sgRNA design tool (http://crispr.mit.edu/), we selected the top six off-target sites overall as well as all seven off-target sites specifically within genes to examine potential off-target effects in three representative mosaic mice that show severe photoreceptor degeneration and/or rhodopsin mislocalization. We did not observe any surveyor nuclease cleavage (Supplementary Fig. S4b) of predicted off-target sites while efficient cleavage of the intended *Kcnj13* site was seen, indicating that our sgRNA targeting is specific.

## Discussion

The CRISPR-Cas9 system can be used to efficiently generate mosaic mice. Consistent with published data^6^,^7^,^19^,^25^, the CRISPR-Cas9 generated mosaicism was commonly detected in two separate zygote injections with a high efficiency of 80% in total: 8 out of 10 examined *Kcnj13* F0 mutant mice showed KCNJ13 mosaic expression. These animals showed different ranges of KCNJ13 mosaic expression; of particular note, KCNJ13 expression was not observed in k713110103 RPE cells (Fig. 3e) although one wild-type allele was detected in F1 progeny together with 3 other mutant alleles (Fig. 1). The highly variable degrees of mosaic KCNJ13 expression are consistent with CRISPR-Cas9 generated pigmentation mosaicism following targeting of the *Tyrosinase* gene^25^. Moreover, the range of mosaic KCNJ13 expression in the eye could be roughly inferred by tail DNA genotyping results. In this study, three F0 mutant mice (k713110103, k713110104, and k813110115) with four different alleles showed reduced or completely absent KCNJ13 expression compared to other F0 mosaics with two or three alleles, indicating that tail DNA genotyping results approximate retinal mosaicism.

Although CRISPR-Cas9 generated mosaicism was detected in two separate injections with different doses of sgRNAs and *Cas9* mRNA, we recommend the high-dose injection. The low-dose injection resulted in a low mutagenesis efficiency while the high-dose injection showed a high mutagenesis rate and P1 lethality in our hands. Recently, Singh et al. (2014) discussed injection dose and pronuclear versus cytoplasmic RNA injection by combining both published and their own data and concluded that injections of *Cas9* mRNA at 50–150 ng/µl and sgRNA at 50–75 ng/µl yield good survival of embryos and efficient editing by non-homologous end joining in newborns^26^. Therefore, given that germline *Kcnj13* homozygotes and transheterozygotes die at P1, our observed P1 mortality of some F0 animals is likely due to efficient targeting of *Kcnj13* that is achieved by a high-dose injection.

The observation that 100% of germline *Kcnj13* transheterozygotes (and homozygotes) die at P1 suggests that off-target effects are unlikely to be the cause of lethality. In addition, results from 12 potential off-target sites were negative, indicating that our selected sgRNA is specific to *Kcnj13*. These findings strongly suggest that loss of *Kcnj13* function is postnatal lethal in mice; however, it is interesting to note that a homozygous nonsense mutation (p.Arg166X)^17^ in *KCNJ13* in humans causes LCA disease and not death. There are at least two obvious models to explain this difference. First, p.Arg166X may not cause strong or complete loss of KCNJ13 function. However, since wild-type KCNJ13 protein is 360 amino acids in length, this model seems somewhat unlikely. Therefore, a second model where *Kcnj13* has a developmentally important role in mice that is not conserved in humans would appear to be more feasible.

The CRISPR-Cas9 generated *Kcnj13* F0 mosaics allowed us to bypass the P1 lethality and rapidly provided evidence that *Kcnj13* strong loss-of-function alleles can mimic the human LCA disease phenotype. In mouse k713110103, which appears to completely lack *Kcnj13* function in RPE cells, severe loss of photoreceptor cells was observed at 17 weeks of age. Our results support the hypothesis that functional loss of *Kcnj13* in RPE cells causes rapid photoreceptor degeneration early in life. By 13 weeks after birth, rhodopsin mislocalization (Supplementary Fig. S2b) and decreased ERG a- and b-waves (Fig. 2a,b) are observed in mosaic mouse k813110115 with low levels of KCNJ13 expression in RPE cells. Notably, the observed rhodopsin mislocalization occurs mainly in regions without KCNJ13 expression (Supplementary Fig. S2b), suggesting that *Kcnj13* mutant RPE cells contribute to this phenotype. This relationship is further supported by F0 mosaic mouse k713110104 at 33 weeks of age. In this mouse, RPE cells lacking KCNJ13 correlate with rhodopsin mislocalization and loss of photoreceptors while the position of RPE cells with readily detectable KCNJ13 expression coincides with preserved photoreceptors and also provides a good internal positive control (Fig. 3h; Supplementary Fig. S2d). These results demonstrate that loss of *Kcnj13* function leads to retinal pathology and KCNJ13 expression in RPE cells is required for the development and/or maintenance of photoreceptors, although the molecular basis of this function of *Kcnj13* is yet to be investigated.

*Kcnj13* appears to act in a non-cell-autonomous manner and indirectly regulates photoreceptor cell survival. In large patches where KCNJ13 is absent, severe photoreceptor loss is observed (Fig. 3k), while the RPE cell layer still survives as judged by the presence of RPE65 protein (Fig. 3k,m) and the lack of TUNEL staining in RPE mutant cells and the consistent, although rare detection of TUNEL staining in overlying photoreceptor cells. In contrast, if the area of KCNJ13 loss is small and adjacent to a relatively large region of KCNJ13 expression, the corresponding ONL shows similar thickness to the neighboring ONL with normal KCNJ13 expression (Fig. 3h; Supplementary Fig. S2d) and also does not show rhodopsin mislocalization (Supplementary Fig. S2d). These observations suggest that KCNJ13 function is cell non-autonomous and its expression in RPE cells can rescue surrounding photoreceptor cells by some indirect mechanism. It also explains why no significant morphological changes are observed in mosaic mice with more robust, but still patchy KCNJ13 expression (Fig. 3c,f; Supplementary Fig. S2c). Interestingly, if a small patch of KCNJ13 expression is located as an “island” in a large area otherwise lacking KCNJ13 expression, its ability to rescue neighboring photoreceptors is limited. In such regions, rhodopsin mislocalization is observed and the ONL is thin (Fig. 3i; Supplementary Fig. S2d, marked by an asterisk). KCNJ13, an inwardly rectifying potassium channel, is likely to regulate K^+^ transport and play a role in the maintenance of sub-retinal K^+^ concentration^16^. Alternatively, KCNJ13, which is expressed at the apical membrane of RPE cells^14^,^16^, may help maintain the balance of certain molecules in the subretinal space. It is therefore understandable that a small region of KCNJ13 expression may not be functionally capable of maintaining a large patch of photoreceptor survival. Our results show that pattern of KCNJ13 expression correlates well with the loss of photoreceptor cells and that loss of *Kcnj13* function likely leads to photoreceptor degeneration by an indirect mechanism.

Although it will be necessary to generate a conditional knockout mouse model to fully investigate the detailed mechanism of *Kcnj13*-associated photoreceptor degeneration, the *Kcnj13* F0 mosaics reported here have allowed us to rapidly test if the mouse will provide a good model for studying *KCNJ13*-associated LCA disease phenotypes. Since complete loss of *Kcnj13* likely causes postnatal lethality, KCNJ13 is not detectably expressed in RPE cells until after P1, and no morphological differences in the retina are observed between *Kcnj13* null mutant and wild-type P1 mice (data not shown), it became necessary to make a conditional loss-of-function *Kcnj13* model to study this gene in the adult. One commonly used approach to this end is to introduce two *loxP* sequences flanking an essential exon(s) in the gene of interest and then induce subsequent tissue-specific Cre-mediated deletion. Compared to the commonly used *Cre-loxP* approach, CRISPR-Cas9 can produce mosaic mice in an order of magnitude less time and with a similar savings in cost. In addition, CRISPR-Cas9 generated mosaicism often juxtaposes wild-type and mutant patches of cells, providing a good internal positive control for phenotypic analyses. Recently reported Cre-dependent Cas9-expressing mice can also provide tissue-specific ablation of target genes by crosses with mice expressing tissue-specific Cre recombinase and subsequent virus/particle mediated delivery of sgRNA^27^. In addition, these Cas9 mice can avoid the limitation that our engineered mosaicism is present only in founder mice. However, Cre-dependent Cas9-expressing mice, which require tissue-specific and highly expressed Cre and highly efficient delivery of sgRNA, are unlikely to generate distinct but random patches (i.e., clones) of juxtaposed mutant and wild-type cells as observed in our mosaic mice. Moreover, Cre-dependent Cas9-expressing mice are unlikely to be of great utility to study gene function early in embryonic development. Therefore, both methods have an important place in the mouse geneticist's toolkit to uncover gene function, and the choice will be dictated by the timing and particulars of each experiment.

In summary, with one-step zygote injection, CRISPR-Cas9 engineered mosaicism has rapidly revealed that loss of *Kcnj13* function mimics human LCA phenotypes in mice. This approach provides patches of mutant and wild-type cells in the same tissue and enables us to distinguish cell-autonomous versus non-cell-autonomous gene function, thereby providing clues for the design of future studies of the mechanism of gene function. With the rapid increase in the volumes of next-generation sequencing data from patients and model systems, more and more candidate disease genes have been and will be identified. The CRISPR-Cas9 system will greatly accelerate the validation/elucidation of candidate gene function in biology and disease with its many newly discovered applications^28^,^29^, including CRISPR-Cas9 generated mosaicism.

## Methods

### Production of sgRNA and *Cas9* mRNA

The murine *Kcnj13* start codon was selected as a target site to make *Kcnj13* null alleles based on the characteristics of Cas9-nuclease mutagenesis. As shown in Fig. 1, the start codon (blue ATG) contains the potential cleavage site (red arrowhead). The underlined sgRNA target sequence (CAATTACTGCTGTCCATCGC) was checked by a CRISPR design tool (http://crispr.mit.edu/)^30^ and shows very high specificity. All potential off-targets have at least 3 mismatches and also at least one mismatch within the seed region (the 10 bases at the 3′ end of the sgRNA target sequence), predicting low off-target effects based on recent off-target study results^20^,^31^. The murine *Kcnj13* sgRNA sequence was cloned into pDR274 (a sgRNA cloning vector from Addgene, Cambridge MA) to form a T7 promoter-mediated sgRNA expression vector^32^. With *Dra*I digestion, the linearized expression vector was purified using a QIAquick Gel Purification Kit (QIAGEN) and used as a DNA template to produce sgRNA by a MAXIscript T7 kit (Life Technologies). For *Cas9* mRNA production, px330 (from the Zhang Laboratory)^1^ was modified with a T7 promoter at the 5′ end of the *Cas9* coding sequence. The modified px330 vector was digested with *Not*I and then purified using a QIAquick Gel Purification Kit (QIAGEN). Subsequently, the linearized and purified vector was used as a DNA template to produce *Cas9* mRNA using an mMESSAGE mMACHINE T7 Ultra Kit (Life Technologies). sgRNA and *Cas9* mRNA were purified using RNA Clean & Concentrator™-25 (ZYMO Research) and dissolved in RNase-free water. RNA concentrations were measured using a NanoDrop ND1000. Finally, 2 µl each of sgRNA and *Cas9* mRNA were mixed with an equal volume of formamide, respectively, and the denatured mixtures were run on a DNA agarose gel to evaluate RNA quality.

### Injection of sgRNA and *Cas9* mRNA into mouse zygotes

According to the literature^5^, higher concentrations of sgRNA and *Cas9* mRNA generate mutations with higher efficiency than lower concentrations; however, the use of lower concentrations can decrease off-target effects^20^. Therefore, two sgRNA and *Cas9* mRNA mixtures (one containing 50 ng/µl sgRNA + 100 ng/µl *Cas9* mRNA and another containing a 4-fold dilution) were prepared (Supplementary Fig. S1b) using RNase-free water. RNA was microinjected into the cytoplasm of C57BL/6 inbred zygotes. After injection, surviving zygotes were immediately transferred into oviducts of ICR albino pseudopregnant females. All mice were maintained under cycles of 12-hours light and 12-hours dark. All animal operations were approved by the Institutional Animal Care and Use Committee at Baylor College of Medicine and were performed in accordance with relevant guidelines and regulations.

### *Kcnj13* mutation analysis

Mouse genomic DNA was obtained from tail biopsies using the sodium hydroxide extraction method and used for PCR amplification of the CRISPR-Cas9 targeting site (Fig. 1; Supplementary Fig. S1a). After PCR amplification, surveyor assays were performed for all F0 mouse PCR products using the SURVEYOR Mutation Detection Kit (Transgenomic Inc.) to screen F0 mutant mice. Screened F0 mutant mice were further tested by RFLP analysis (Supplementary Fig. S1a,c). To fully characterize mutant alleles in F0 mutant mice, PCR products were cloned using the TOPO TA Cloning Kit (Life Technologies) and eight clones for each F0 mutant mouse were sequenced. For three F0 mutant mice (Table 1), DNA was also extracted from paraffin embedded eye tissue. Briefly, 10 sections (10 µm thick) from each eye were collected and processed with 1 ml xylene to remove paraffin. Tissues were washed by ethanol and incubated at room temperature until all residual ethanol had evaporated. Tissues were then digested by proteinase K and DNA was purified by phenol/chloroform and finally dissolved in 30 µl of water for PCR amplification. For these three F0 mutant mice, PCR sub-cloning and sequencing was performed for tail and eye section DNA and 48–82 random clones were picked for sequencing to compare genotypes with the observed mosaic expression of KCNJ13. For mice showing disease phenotypes, we selectively examined off-target effects using tail and/or eye section DNA using the Surveyor assay (Supplementary Fig. S4) to test for specificity of our *Kcnj13* targeting.

### Dark-adapted ERG analysis

Mice were dark-adapted overnight and then anesthetized by a single intraperitoneal injection of 22 mg/kg ketamine, 4.4 mg/kg xylazine and 0.37 mg/kg acepromazine. Pupils were dilated with a drop of tropicamide (1.0%) and phenylephrine (2.5%) and then corneas were anesthetized with a drop of proparacaine (1.0%). After 1 min, excess fluid was removed and a drop of hypromellose (2%) was placed on each cornea to keep it moistened and provide a good contact between the cornea and the ERG electrode (N1530NNC, LKC Technologies). All tests were performed under a dim red light and a feedback-controlled heating pad was used to keep treated mice at a constant body temperature of 37.6°C. ERG recordings were performed using a UTAS Visual Diagnostic System and EMWIN software (LKC Technologies, Gaithersburg, MD, USA) and six flash intensities (-34, -24, -14, -4, 0 and 10dB) were used. ERG data were plotted by GraphPad Prism5 software (GraphPad Software, La Jolla, CA, USA). Of 11 F0 mutant mice, nine were subjected to ERG tests: three were tested at 13 weeks, four at 22 weeks and two at 33 weeks of age. At each ERG test, F0 wild-type mice were used as controls to evaluate whether F0 mutant mice had decreased responses to light.

### Histological analysis and Immunofluorescence

After ERG tests, F0 mutant and wild-type mice were sacrificed and eyes were fixed in Davidson's buffer^33^ and processed for paraffin embedding. Serial sections (5 µm thick) were cut for approximately one-half of each eye and were processed for H&E and IF staining. For IF staining, antigen retrieval was performed by boiling the sections in TEG buffer (10 mM Trizma base, 0.5 mM EGTA, pH 9.0) for 30 minutes. After cooling at room temperature, sections were permeabilized by incubation in 0.5% TBS-Triton X100 for 20 minutes. Sections were then incubated in 5% donkey serum (in TBS buffer) at room temperature for 1 hour and incubated with primary antibodies (1:50 goat anti-KCNJ13, C19, from Santa Cruz Biotechnology, 1:50 mouse anti-RPE65, E5, from Santa Cruz Biotechnology and 1:300 mouse anti-rhodopsin, B6-30N, a generous gift from W. Clay Smith) overnight at 4°C. After washes in TBS, sections were then incubated with secondary antibodies (1:500 Alexa Fluor® 488 conjugated donkey anti-goat IgG and 1:500 Cy3® donkey anti-mouse IgG, Jackson ImmunoResearch Laboratories) at room temperature for 1 hour, followed by washes and counterstaining of nuclei using DAPI. Finally, slides were mounted with ProLong® Gold antifade reagents (Life Technologies). To check cell death in RPE cells, TUNEL staining was performed with the In Situ Cell Death Detection Kit, TMR red (Roche) following the kit-provided procedures. Images were captured using a fluorescence microscope (Zeiss Axio Imager M2m).

## Author Contributions

H.Z., R.C. and G.M. designed the research. H.Z., Y.C. and Y.L. performed the experiments. H.Z. and G.M. wrote the manuscript. All authors have read and edited the manuscript before submission.

## Supplementary Material

Supplementary Information Supplementary data

## Figures and Tables

**Table 1:** Mutation detection by PCR sub-cloning and sequencing of tail and retinal section DNA

	Detected Genotype Frequency		
Mouse ID	Genotype	Tail	Retinal Section
k713110103	N2	45.1% (37/82)	24.7% (18/73)
	N6	45.1% (37/82)	61.6% (45/73)
	N13	9.8% (8/82)	13.7% (10/73)
k713110104	W4	2.1% (1/48)	4.3% (2/47)
	N7	83.3% (40/48)	70.2% (33/47)
	N13	8.3% (4/48)	23.4% (11/47)
	N15	6.3% (3/48)	2.1% (1/47)
k713110114	N2	54.4% (31/57)	45.5% (25/55)
	W4	45.6% (26/57)	54.5% (30/55)

## References

[b1] Cong L. *et al.* Multiplex genome engineering using CRISPR/Cas systems. Science 339, 819–23 (2013). 23287718 10.1126/science.1231143 PMC3795411

[b2] Mali P. *et al.* RNA-guided human genome engineering via Cas9. Science 339, 823–6 (2013). 23287722 10.1126/science.1232033 PMC3712628

[b3] Jinek M. *et al.* RNA-programmed genome editing in human cells. Elife 2, e00471 (2013). 23386978 10.7554/eLife.00471 PMC3557905

[b4] Cho S. W., Kim S., Kim J. M. & Kim J. S. Targeted genome engineering in human cells with the Cas9 RNA-guided endonuclease. Nat Biotechnol 31, 230–2 (2013). 23360966 10.1038/nbt.2507

[b5] Wang H. *et al.* One-step generation of mice carrying mutations in multiple genes by CRISPR/Cas-mediated genome engineering. Cell 153, 910–8 (2013). 23643243 10.1016/j.cell.2013.04.025 PMC3969854

[b6] Yang H. *et al.* One-step generation of mice carrying reporter and conditional alleles by CRISPR/Cas-mediated genome engineering. Cell 154, 1370–9 (2013). 23992847 10.1016/j.cell.2013.08.022 PMC3961003

[b7] Li D. *et al.* Heritable gene targeting in the mouse and rat using a CRISPR-Cas system. Nat Biotechnol 31, 681–3 (2013). 23929336 10.1038/nbt.2661

[b8] Nakamura K. *et al.* Generation of muscular dystrophy model rats with a CRISPR/Cas system. Sci Rep 4, 5635 (2014). 25005781 10.1038/srep05635 PMC4088098

[b9] Xue W. *et al.* CRISPR-mediated direct mutation of cancer genes in the mouse liver. Nature 514, 380–4 (2014). 25119044 10.1038/nature13589 PMC4199937

[b10] Schwank G. *et al.* Functional repair of CFTR by CRISPR/Cas9 in intestinal stem cell organoids of cystic fibrosis patients. Cell Stem Cell 13, 653–8 (2013). 24315439 10.1016/j.stem.2013.11.002

[b11] Wu Y. *et al.* Correction of a genetic disease in mouse via use of CRISPR-Cas9. Cell Stem Cell 13, 659–62 (2013). 24315440 10.1016/j.stem.2013.10.016

[b12] Yin H. *et al.* Genome editing with Cas9 in adult mice corrects a disease mutation and phenotype. Nat Biotechnol 32, 551–3 (2014). 24681508 10.1038/nbt.2884 PMC4157757

[b13] Yoshimi K., Kaneko T., Voigt B. & Mashimo T. Allele-specific genome editing and correction of disease-associated phenotypes in rats using the CRISPR-Cas platform. Nat Commun 5, 4240 (2014). 24967838 10.1038/ncomms5240 PMC4083438

[b14] Kusaka S. *et al.* Functional Kir7.1 channels localized at the root of apical processes in rat retinal pigment epithelium. J. Physiol. 531, 27–36 (2001). 11179389 10.1111/j.1469-7793.2001.0027j.x PMC2278447

[b15] Zhang W. *et al.* Characterization of the R162W Kir7.1 mutation associated with snowflake vitreoretinopathy. Am. J. Physiol. Cell Physiol. 304, C440–C449 (2013). 23255580 10.1152/ajpcell.00363.2012 PMC3602648

[b16] Yang D., Pan A., Swaminathan A., Kumar G. & Hughes B. A. Expression and localization of the inwardly rectifying potassium channel Kir7.1 in native bovine retinal pigment epithelium. Invest Ophthalmol Vis Sci 44, 3178–85 (2003). 12824269 10.1167/iovs.02-1189

[b17] Sergouniotis P. I. *et al.* Recessive mutations in KCNJ13, encoding an inwardly rectifying potassium channel subunit, cause leber congenital amaurosis. Am J Hum Genet 89, 183–90 (2011). 21763485 10.1016/j.ajhg.2011.06.002 PMC3135807

[b18] den Hollander A. I., Roepman R., Koenekoop R. K. & Cremers F. P. Leber congenital amaurosis: genes, proteins and disease mechanisms. Prog Retin Eye Res 27, 391–419 (2008). 18632300 10.1016/j.preteyeres.2008.05.003

[b19] Sung Y. H. *et al.* Highly efficient gene knockout in mice and zebrafish with RNA-guided endonucleases. Genome Res 24, 125–31 (2014). 24253447 10.1101/gr.163394.113 PMC3875853

[b20] Fu Y. *et al.* High-frequency off-target mutagenesis induced by CRISPR-Cas nucleases in human cells. Nat Biotechnol 31, 822–6 (2013). 23792628 10.1038/nbt.2623 PMC3773023

[b21] Rossant J. & Spence A. Chimeras and mosaics in mouse mutant analysis. Trends Genet 14, 358–63 (1998). 9769731 10.1016/s0168-9525(98)01552-2

[b22] Gao J. *et al.* Progressive photoreceptor degeneration, outer segment dysplasia, and rhodopsin mislocalization in mice with targeted disruption of the retinitis pigmentosa-1 (Rp1) gene. Proc Natl Acad Sci U S A 99, 5698–703 (2002). 11960024 10.1073/pnas.042122399 PMC122834

[b23] Hamel C. P. *et al.* A developmentally regulated microsomal protein specific for the pigment epithelium of the vertebrate retina. J Neurosci Res 34, 414–25 (1993). 8474143 10.1002/jnr.490340406

[b24] Chen Y., Moiseyev G., Takahashi Y. & Ma J. X. RPE65 gene delivery restores isomerohydrolase activity and prevents early cone loss in Rpe65-/- mice. Invest Ophthalmol Vis Sci 47, 1177–84 (2006). 16505056 10.1167/iovs.05-0965

[b25] Yen S. T. *et al.* Somatic mosaicism and allele complexity induced by CRISPR/Cas9 RNA injections in mouse zygotes. Dev Biol 393, 3–9 (2014). 24984260 10.1016/j.ydbio.2014.06.017 PMC4166609

[b26] Singh P., Schimenti J. C. & Bolcun-Filas E. A Mouse Geneticist's Practical Guide to CRISPR Applications. Genetics, 10.1534/genetics.114.169771 (2014). PMC4286675 25271304

[b27] Platt R. J. *et al.* CRISPR-Cas9 Knockin Mice for Genome Editing and Cancer Modeling. Cell 159, 440–55 (2014). 25263330 10.1016/j.cell.2014.09.014 PMC4265475

[b28] Zhang F., Wen Y. & Guo X. CRISPR/Cas9 for genome editing: progress, implications and challenges. Hum Mol Genet 23, R40–6 (2014). 24651067 10.1093/hmg/ddu125

[b29] Hsu P. D., Lander E. S. & Zhang F. Development and applications of CRISPR-Cas9 for genome engineering. Cell 157, 1262–78 (2014). 24906146 10.1016/j.cell.2014.05.010 PMC4343198

[b30] Hsu P. D. *et al.* DNA targeting specificity of RNA-guided Cas9 nucleases. Nat Biotechnol 31, 827–32 (2013). 23873081 10.1038/nbt.2647 PMC3969858

[b31] Cradick T. J., Fine E. J., Antico C. J. & Bao G. CRISPR/Cas9 systems targeting beta-globin and CCR5 genes have substantial off-target activity. Nucleic Acids Res 41, 9584–92 (2013). 23939622 10.1093/nar/gkt714 PMC3814385

[b32] Hwang W. Y. *et al.* Efficient genome editing in zebrafish using a CRISPR-Cas system. Nat Biotechnol 31, 227–9 (2013). 23360964 10.1038/nbt.2501 PMC3686313

[b33] Latendresse J. R., Warbrittion A. R., Jonassen H. & Creasy D. M. Fixation of testes and eyes using a modified Davidson's fluid: comparison with Bouin's fluid and conventional Davidson's fluid. Toxicol Pathol 30, 524–33 (2002). 12187944 10.1080/01926230290105721

