# Supplementary material for: CRISPR-engineered mosaicism rapidly reveals that loss of Kcnj13 function in mice mimics human disease phenotypes
Source: Sci Rep. 2015 Feb 10;5:8366. doi: 10.1038/srep08366 (PMC4322368; doi:10.1038/srep08366)
Supplement: Supplementary Information — Supplementary data [file srep08366-s1.pdf]

Supplementary Information

CRISPR-engineered mosaicism rapidly reveals that loss of *Kcnj13* function in mice mimics human disease phenotypes

Hua Zhong[1], Yiyun Chen[2,3], Yumei Li[2,3], Rui Chen[2,3,4], and Graeme Mardon[1,3,4,5,6]

[1]Department of Pathology and Immunology, Baylor College of Medicine, Houston, TX 77030, [2]HGSC, Baylor College of Medicine, Houston, TX 77030, [3]Department of Molecular and Human Genetics, Baylor College of Medicine, Houston, TX 77030, [4]Program in Developmental Biology, Baylor College of Medicine, Houston, TX 77030, [5]Department of Neuroscience, Baylor College of Medicine, Houston, TX 77030, [6]Department of Ophthalmology, Baylor College of Medicine, Houston, TX 77030.

Correspondence and requests for materials should be addressed to G.M. (email: gmardon@bcm.edu)

Figure Legends

Supplementary Figure S1. sgRNA design, zygote injection, and genotyping. (**a**) Schematic of *Kcnj13* gene structure and sgRNA target sequence information. A restriction enzyme site (*BtgZ*I) is located at the putative cleavage site of Cas9 nuclease and provides an opportunity to check CRISPR-Cas9 produced mutations by PCR-RFLP analysis. (**b**) sgRNA and *Cas9* mRNA concentrations used for zygote injection and F0 founder mouse production data. (**c**) PCR-RFLP analysis to confirm F0 mutant mice. All F0 mice failed to show complete cleavage by restriction enzyme *BtgZ*I compared to a wild-type control, indicating that CRISPR-Cas9 efficiently produced mutations at the selected target site.

Supplementary Figure S2. CRISPR-Cas9-mediated KCNJ13 F0 mosaic expression in whole retinal sections and corresponding morphological phenotypes. (**a**) KCNJ13 expression in RPE cells from a 13-week-old F0 wild-type mouse. (**b**) Observed KCNJ13 mosaic expression and rhodopsin mislocalization in F0 mosaic mouse k813110115 at 13 weeks of age following CRISPR-Cas9 zygote injection. (**c**) A representative section showing somewhat reduced KCNJ13 expression in F0 mosaic mouse k713110113 at 22 weeks of age. Little or no photoreceptor cell loss is observed. (**d**) Patchy KCNJ13 expression, loss of photoreceptor cells, and rhodopsin mislocalization observed in F0 mosaic mouse k713110104 at 33 weeks of age. Cell non-autonomous rescue of photoreceptor cells by surrounding KCNJ13-expressing RPE cells is shown in the inset. White arrowheads mark rhodopsin mislocalization.

Supplementary Figure S3. No programmed cell death is observed in the RPE cell layer in the absence of *Kcnj13* function. A representative retinal section of mosaic mouse k713110104 is shown. Although we observed rare TUNEL-positive cells in the ONL only in regions showing photoreceptor loss (indicated by DPAI staining), no TUNEL staining was observed in the RPE. The observed TUNEL signals marked by white arrowheads are shown in the insets. RPE, retinal pigment epithelium. ONL, outer nuclear layer. INL, inner nuclear layer.

Supplementary Figure S4. Off-target effects are not observed in the top predicted sites using the Surveyor assay. (**a**) We examined the top 6 predicted off-target sites overall (off-target #6, 8-12) and all 7 gene-related off-target sites (off-target #1-7), of which off-target #6 is predicted to be the second most probable off-target site overall. (**b**) Surveyor nuclease cleavages are clearly observed only at the intended *Kcnj13* on-target site and not in any other examined off-target site.

a

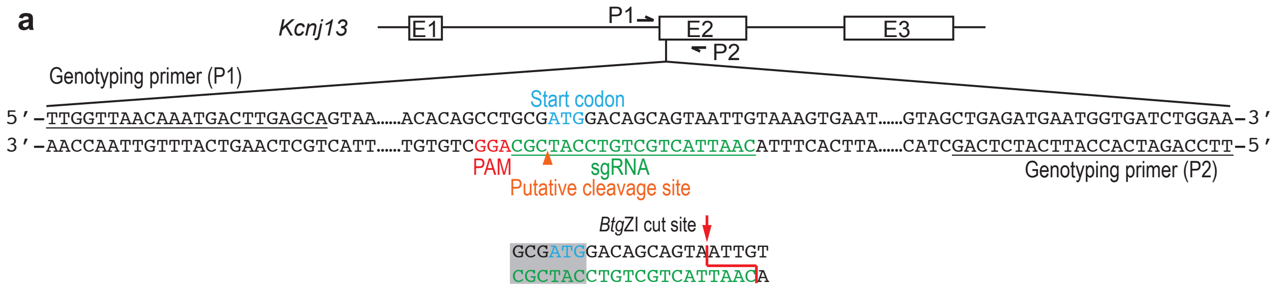

b

sgRNA & Cas9 mRNA concentration	Injected zygotes	Transferred zygotes	Newborns	Survived Pups
High concentration: 50 ng/µl + 100 ng/µl	220	140	28	8
Low concentration: 12.5 ng/µl + 25 ng/µl	300	129	39	38

c

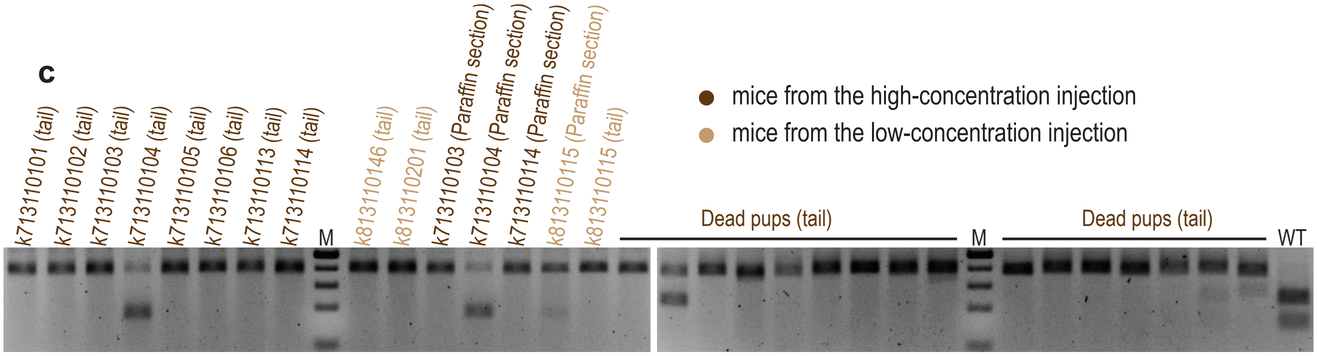

Supplementary Figure S1

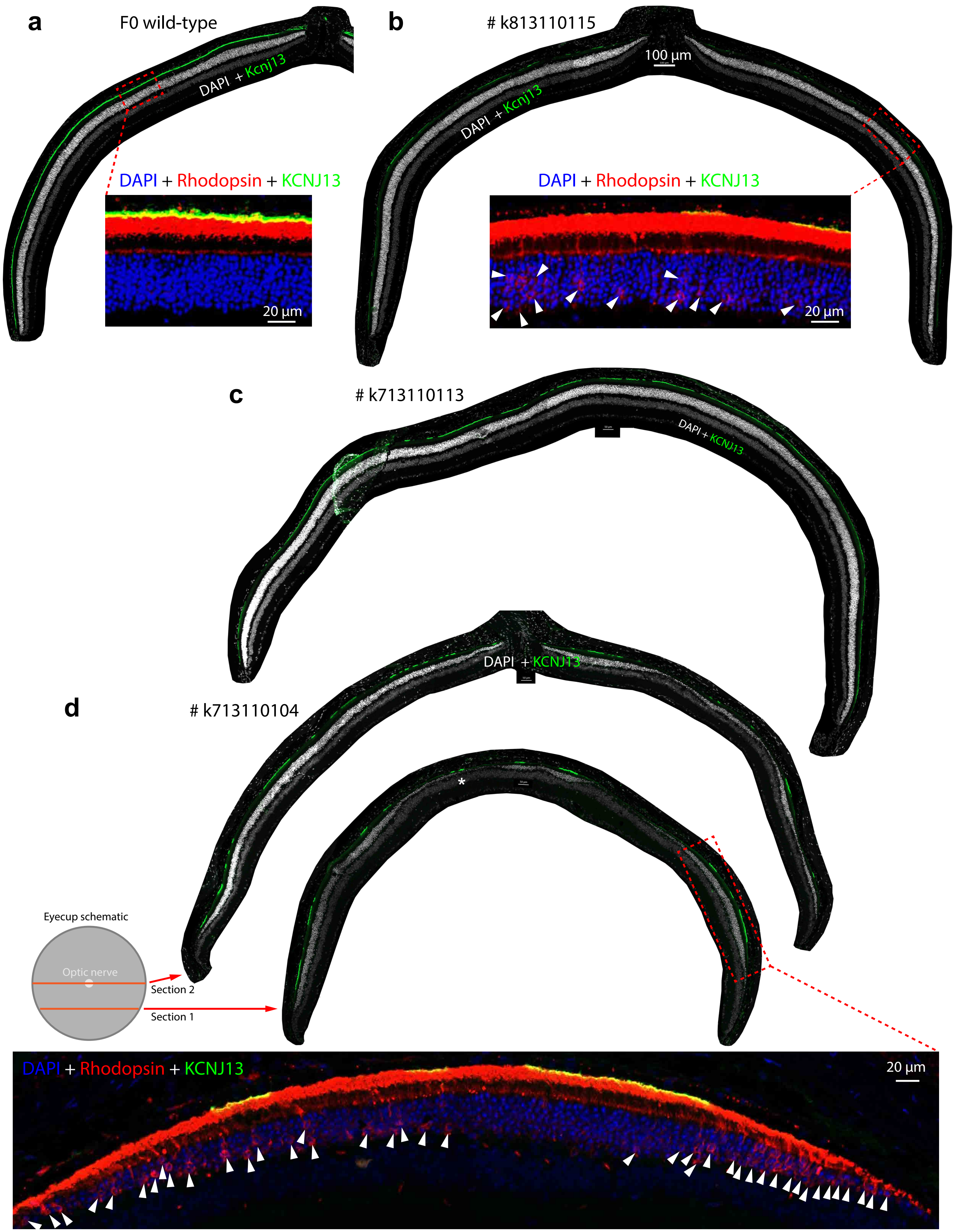

Supplementary Figure S2

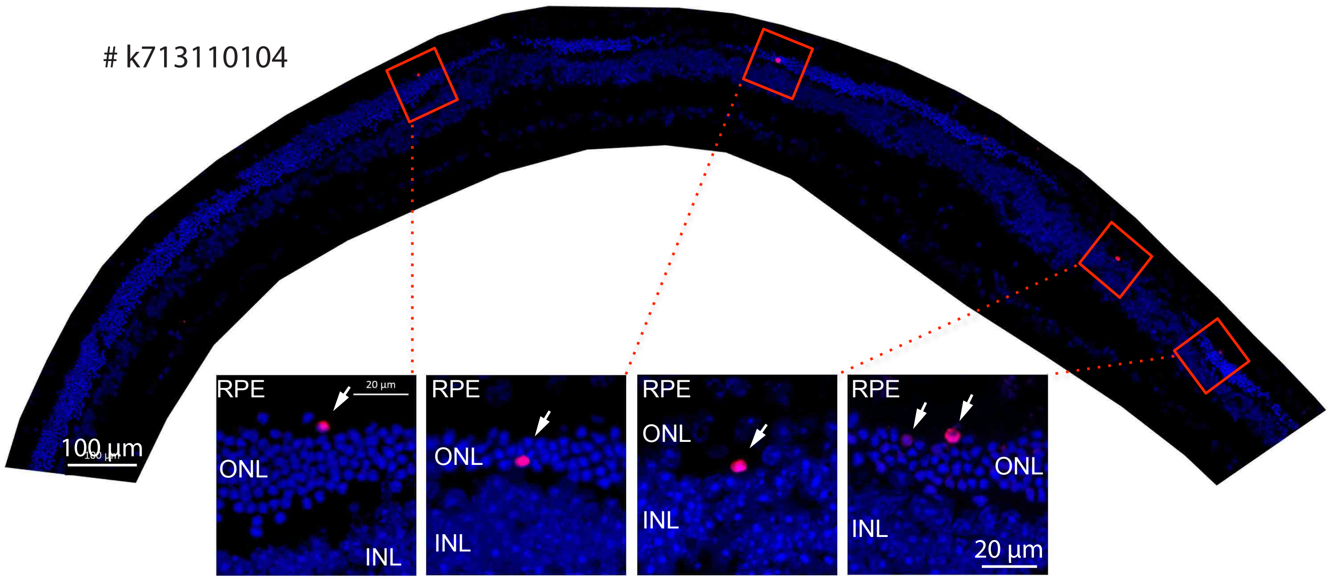

Supplementary Figure S3

a

Target ID	Sequence	Score	mismatches	UCSC gene	Locus	Genotyping primers
On-target	CAATTACTGCTGTCCATCGCAGG	87		NM_001172417	chr1:-89285947	TTGGTTAACAAATGACTTGAGCA; TTCCAGATCACCATTCATCTCAG
Off-target #1	CACTTACTGGAGTCCATTGCAGG	0.2	4MMs [3:10:11:18]	NM_001177600	chr1:-63575394	TGAGTTTTCTGCAAAATCTAGGTG; CCATTGGTGGCCTTTCTCT
Off-target #2	CAAGTGCTGCTTTCCATCGAAGG	0.2	4MMs [4:6:12:20]	NM_172938	chr10:-42654763	CACCTTGGGAGACAGCATCT; GGCGAGACTTCCAGTGTGAT
Off-target #3	CACCTACTGCTGGCCATCTCAAG	0.2	4MMs [3:4:13:19]	NM_199299	chr11:+51634727	TCCGTGAGAAGGAACCTGAC; GTTTCGGGGTGAATTCTCAA
Off-target #4	CAGTTGCTTCTGTCCATCTCTGG	0.2	4MMs [3:6:9:19]	NM_177780	chr14:+68402805	ATTAAGATGTCGGGCAGGAA; GGATCTGGCATCCTCTTGTG
Off-target #5	CAGTGAGTGCTGTCCATTGCTGG	0.2	4MMs [3:5:7:18]	NM_133365	chr15:+28226833	TTGTGGCTGGTAATGTCTGG; GAGGAGTTGGTGGATGGGTA
Off-target #6	GAGTTACTGCTGTCCATTGCAAG	0.7	3MMs [1:3:18]	NM_009185	chr4:-114681978	AACCCACTTTCCCTAAAAGGAG; TTCCTCAGGCTTTACAATACCA
Off-target #7	CAATCATTGCTGTCTATAGCTGG	0.1	4MMs [5:7:15:18]	NM_027758	chr8:+85773229	ACCGAGAGAGGGAAGGTCAC; GGGATTGCGAAAAGCATAAG
Off-target #8	GAATTCCTGCTGTCCATCTCTGG	0.7	3MMs [1:6:19]		chr2:+14850128	AAACGTCCTGTGCACTGTGT; TGACAGCCCAGAGACTGTTG
Off-target #6	GAGTTACTGCTGTCCATTGCAAG	0.7	3MMs [1:3:18]	NM_009185	chr4:-114681978	
Off-target #9	TACTTACTGTTGTCCATCGATGG	0.6	4MMs [1:3:10:20]		chr15:+61625838	GGCTTGACAGCCATACATGA; AGCCAAGGATCTCAGGAGTG
Off-target #10	CAGTTACAGCTGTCCATGGCTGG	0.6	3MMs [3:8:18]		chr12:+30046463	ATGCCCGCCAGTAAGATAGA; AGACAAGTGGGGGACTTGAA
Off-target #11	CAAATTCTGCTGTCCATCTCTGG	0.6	3MMs [4:6:19]		chrX:+50469308	GTCAGCTCACAGGGAAAAGG; GGCAGAGTTAGGTGACAGCA
Off-target #12	CAACTCCTGCTGTCCATCTCCAG	0.6	3MMs [4:6:19]		chr11:-34111355	CTGCCACATGGCTTAGAACA; CTGAGGGTGCGAATTGTAGC

b

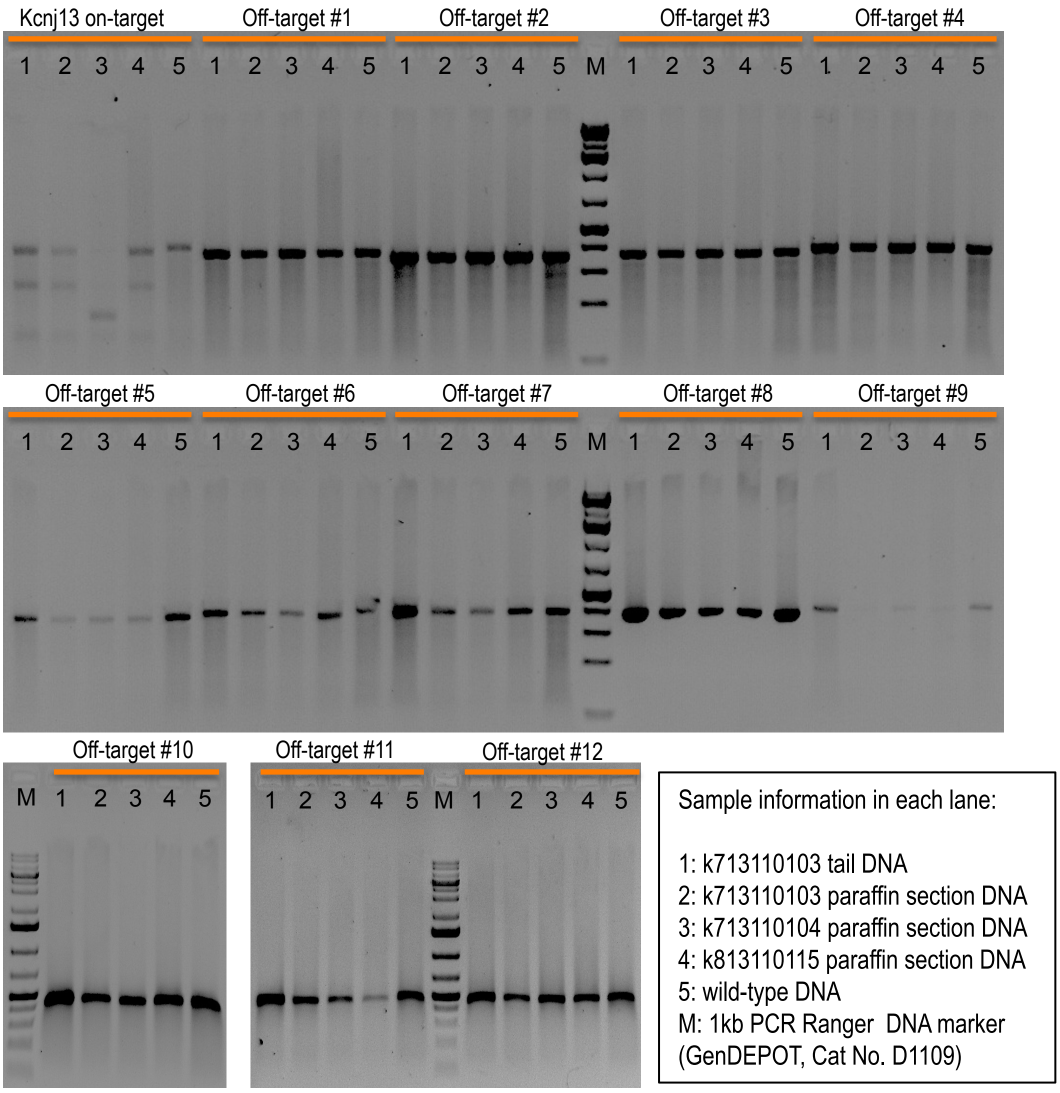

Sample information in each lane:

1: k713110103 tail DNA
2: k713110103 paraffin section DNA
3: k713110104 paraffin section DNA
4: k813110115 paraffin section DNA
5: wild-type DNA
M: 1kb PCR Ranger DNA marker (GenDEPOT, Cat No. D1109)

Supplementary Figure S4
